# Methylmercury and the Brain: Griffiths et al. Respond

**DOI:** 10.1289/ehp.10302R

**Published:** 2007-08

**Authors:** Charles Griffiths, Al McGartland, Maggie Miller

**Affiliations:** National Center for Environmental Economics, U.S. Environmental Protection Agency, Washington, DC, E-mail: griffiths.charles@epa.gov

In our review of the article by Trasande et al. (2005), we used their published linear model to evaluate the monetized impact of IQ decrements associated with prenatal mercury exposure to methylmercury (MeHg) under different assumptions (Griffiths et al. 2007). First, we used a corrected dose–response slope to address the error that the authors made in the conversion of the relationship between cord blood and neurodevelopment effects. We then introduced the assumptions that the U.S. Environmental Protection Agency (EPA) used in its Clean Air Mercury Rule (CAMR). Introducing the U.S. EPA assumptions decreased the undiscounted monetized impact of global anthropogenic mercury emissions in the corrected Trasande et al. model by 81% and decreased the estimated impact of U.S. sources by almost 97%. When discounting is included, the U.S. EPA assumptions decreased the monetized estimate of global impacts by 88% and the impact of U.S. power plants by 98%.

The choice of a linear model (i.e., a K-power model, with *K* = 1) was based on the recommendation of the National Research Council (NRC 2000):

After extensive discussion, the committee concluded that the most reliable and defensible results for the purpose of risk assessment are those based on the K-power model.

Trasande et al. choose to emphasize the results of their logarithmic model, which produces their highest estimates of monetized impacts. We do not dispute that there may be cases in which a logarithmic model might be appropriate, but in the case of methylmercury, the NRC (2000) was unequivocal:

For MeHg, the committee believes that a good argument can be made for the use of a K-power model with K constrained to be greater than or equal to 1. That rules out square-root (K = 0.5) and log models (the limiting case as K approaches 0).

For the U.S. EPA dose–response slope, we used the results of an integrated statistical analysis by Ryan (2005), which has been recently updated (Axelrad et al. 2007). The analysis of Axelrad et al. includes results from the Seychelles study and also those of the Faroe Islands study (which was used by Trasande et al. 2005), as well as the New Zealand study. All three of these studies were used by the NRC (2000) and are described as being “well designed and carefully conducted, and each examined prenatal MeHg exposures within the range of the general U.S. population exposures.” We will concede that controlling for maternal fish intake when assessing the impact of mercury on neurodevelopment is an important consideration that can be addressed in the future.

The assumption that, on average, 16% of the total mercury deposition in the United States is from American and Canadian sources comes straight from the U.S. EPA model used for the CAMR. As discussed in our article (Griffiths et al. 2007), the U.S. EPA used a spatially explicit air quality model to simulate the location of mercury deposition, but we used the average value to compare it to Trasande et al.’s (2005) assumption that 60% of the mercury content in all domestically caught fish is due to American sources. It is true that in the study of Steubenville, Ohio, published after the CAMR was promulgated, Keeler (2006) found a much higher percentage of local and regional deposition (70% of the mercury wet deposition, not 80–90%), but this is an estimate of deposition at a single point and cannot be extrapolated to the entire country. Furthermore, the same U.S. EPA model that produced the 16% average value predicts comparatively high values for the Steubenville region of Ohio (U.S. EPA 2006).

With regard to the charge that we assumed there will be no reductions in fish contamination until after 15 years, Transande et al. are wrong. In our article (Griffiths et al. 2007) we are clear in our position that benefits build over time during the transition path from the current conditions to the new equilibrium. The choice of 15 years is an average period over which to discount the benefits, reflecting the 5–30 years for freshwater systems and the 30–200 years for ocean systems to reach equilibrium. Furthermore, we reported the undiscounted monetized results, which could be compared to Trasande et. al’s (2005) implicit assumption of the instantaneous elimination of all anthropogenic mercury from the environment.

Finally, Trasande et al.’s reference to Viscusi and Aldy (2004) is truly baffling. That article is a review and evaluation of dozens of studies on the value of a statistical life (VSL). A VSL is derived from the tradeoffs witnessed in the market and elsewhere between income and small changes in risk of death. The value for a small change in mortality risk is aggregated to statistical lives in order to be comparable to risk assessment estimates. Because mortality risk and IQ decrements are vastly different items, there is no expected relationship between these two values.
